# Designing efficient randstrobes for sequence similarity analyses

**DOI:** 10.1093/bioinformatics/btae187

**Published:** 2024-04-05

**Authors:** Moein Karami, Aryan Soltani Mohammadi, Marcel Martin, Barış Ekim, Wei Shen, Lidong Guo, Mengyang Xu, Giulio Ermanno Pibiri, Rob Patro, Kristoffer Sahlin

**Affiliations:** Department of Mathematics, Science for Life Laboratory, Stockholm University, Stockholm 106 91, Sweden; Department of Mathematics, Science for Life Laboratory, Stockholm University, Stockholm 106 91, Sweden; Department of Biochemistry and Biophysics, National Bioinformatics Infrastructure Sweden, Science for Life Laboratory, Stockholm University, Solna SE-17121, Sweden; Computer Science and Artificial Intelligence Laboratory (CSAIL), Massachusetts Institute of Technology (MIT), Cambridge, MA 02139, United States; Broad Institute of MIT and Harvard, Cambridge, MA 02142, United States; Department of Infectious Diseases, Key Laboratory of Molecular Biology for Infectious Diseases (Ministry of Education), Institute for Viral Hepatitis, The Second Affiliated Hospital of Chongqing Medical University, Chongqing 400010, China; BGI Research, Qingdao 266555, China; BGI Research, Shenzhen 518083, China; Department of Environmental Sciences, Informatics and Statistics, Ca’ Foscari University of Venice, Venice 30172, Italy; ISTI-CNR, Pisa 56124, Italy; Department of Computer Science and Center for Bioinformatics and Computational Biology, University of Maryland, College Park, MD 20742, United States; Department of Mathematics, Science for Life Laboratory, Stockholm University, Stockholm 106 91, Sweden

## Abstract

**Motivation:**

Substrings of length *k*, commonly referred to as *k*-mers, play a vital role in sequence analysis. However, *k*-mers are limited to exact matches between sequences leading to alternative constructs. We recently introduced a class of new constructs, *strobemers*, that can match across substitutions and smaller insertions and deletions. *Randstrobes*, the most sensitive strobemer proposed in Sahlin (Effective sequence similarity detection with strobemers. Genome Res 2021a;31:2080–94. https://doi.org/10.1101/gr.275648.121), has been used in several bioinformatics applications such as read classification, short-read mapping, and read overlap detection. Recently, we showed that the more pseudo-random the behavior of the construction (measured in entropy), the more efficient the seeds for sequence similarity analysis. The level of pseudo-randomness depends on the construction operators, but no study has investigated the efficacy.

**Results:**

In this study, we introduce novel construction methods, including a Binary Search Tree-based approach that improves time complexity over previous methods. To our knowledge, we are also the first to address biases in construction and design three metrics for measuring bias. Our evaluation shows that our methods have favorable speed and sampling uniformity compared to existing approaches. Lastly, guided by our results, we change the seed construction in strobealign, a short-read mapper, and find that the results change substantially. We suggest combining the two results to improve strobealign’s accuracy for the shortest reads in our evaluated datasets. Our evaluation highlights sampling biases that can occur and provides guidance on which operators to use when implementing randstrobes.

**Availability and implementation:**

All methods and evaluation benchmarks are available in a public Github repository at https://github.com/Moein-Karami/RandStrobes. The scripts for running the strobealign analysis are found at https://github.com/NBISweden/strobealign-evaluation.

## 1 Introduction

In sequence analyses, *k*-mers play an important role in various algorithms and approaches. For example, *k*-mers can be used as *seeds* for sequence similarity search, where a seed shared between two sequences acts as an *anchor* to identify similar regions between, e.g. DNA, RNA, or protein sequences. When used as seeds, *k*-mers enable rapid identification of shared regions and are used in a large number of short and long-read mapping algorithms (Alser *et al.* 2021, Sahlin *et al.* 2023), and other approaches for querying large sequence datasets (Marchet *et al.* 2021, Fan *et al.* 2024).

Both a feature and a limitation of using *k*-mers as seeds is that sequences must be identical for the seed to match. In biological data, it is common that mutations in DNA occur in the form of substituted, deleted, and inserted nucleotides. In addition, common DNA and RNA sequencing techniques are noisy and introduce additional altering of the nucleic acids. In order to provide anchors also in regions with high divergence, seeds are allowed to *anchor* over mutations. Alternatives to *k*-mers have therefore been explored extensively in the literature, such as spaced *k*-mers (Ma *et al.* 2002). See Sahlin *et al.* (2023) for an overview of several other seeding constructs used in read mapping.

### 1.1 Strobemers

**Figure 1.**
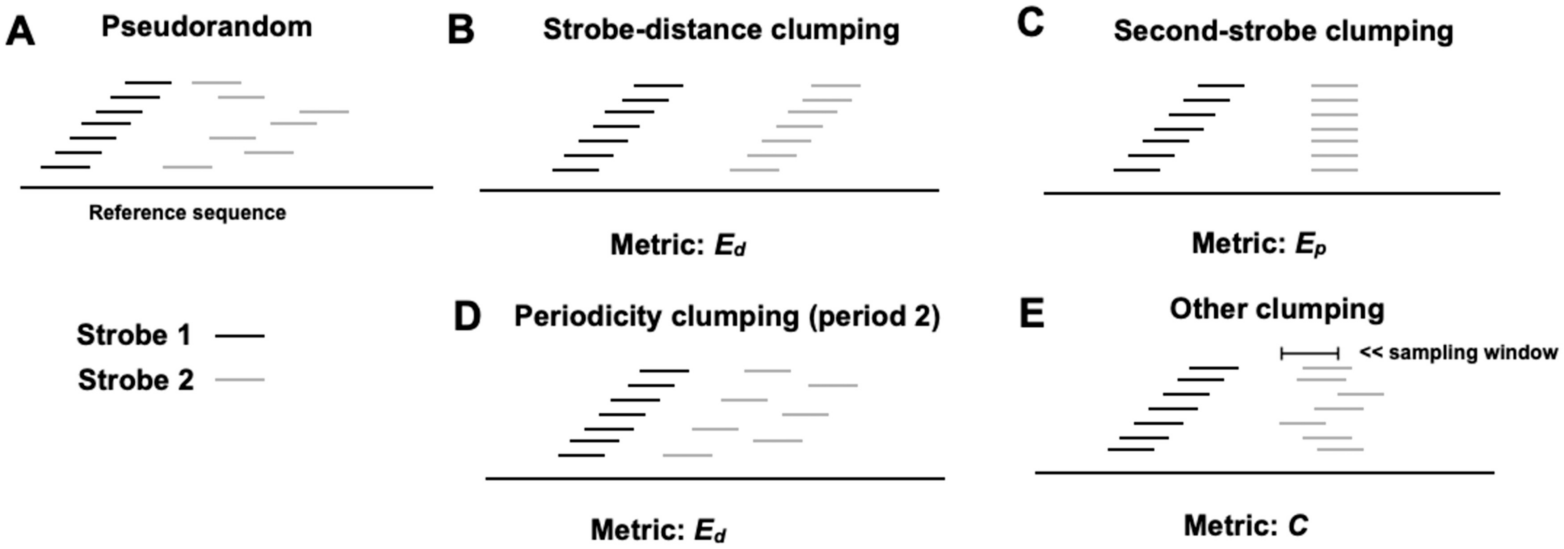
Illustration of a desired random sampling of the second strobe for strobemers consisting of two strobes (case A). Whenever a pseudo-random method is used to select the downstream strobe based on the first strobe, it generates some sampling bias. Cases B–E show different biases we observed in the sampling. The metrics we propose to measure the bias are displayed under each of the illustrations of cases B–E.

Recently, we introduced *strobemers*, a novel class of seed constructs (Sahlin 2021a). Strobemers can produce seed matches across substitutions, insertions, and deletions, expanding on ideas from neighboring minimizer pairs (Chin and Khalak 2019, Sahlin and Medvedev 2021) and *k*-min-mers (Ekim *et al.* 2021) that link neighboring minimizers (Roberts *et al.* 2004) into a seed. Strobemers generalize this linking by considering downstream *k*-mers as potential candidates to link, offering various methods such as minstrobes, randstrobes, and hybridstrobes (Sahlin 2021a), with *randstrobes* being the most effective. Randstrobes have been used, e.g. in for short-read mapping (Sahlin 2022), transcriptomic long-read normalization (Nip *et al.* 2023), and read classification (Xu *et al.* 2023). Our recent study also demonstrates that randstrobes provide accurate sequence similarity ranking using the Jaccard distance (Maier and Sahlin 2023). This study also revealed a strong correlation between strobemers’ sensitivity and the pseudo-randomness of the seed construct, measured through entropy (Maier and Sahlin 2023). While additional strobemer variants have been introduced (Maier and Sahlin 2023), randstrobes remain the simplest and most widely used construct. Constructing randstrobes involves converting strings to integers using a hash function and selecting candidate *k*-mers for linking through a link function and comparator operator. Sampling biases (Fig. 1) in this process can affect sequence matching efficiency (Maier and Sahlin 2023). So far, the underlying operators to produce randstrobes have not been evaluated.

### 1.2 Our contribution

We design metrics suitable for detecting and measuring several types of bias in randstrobe construction methods (Fig. 1). Using the new evaluation metrics, we uncovered biases and limitations in previous randstrobe methods (Sahlin 2021a, 2022, Xu *et al.* 2023). We propose new methods to enhance the core operations (hashing, linking, and comparison), which improve seed uniqueness, sampling uniformity, and construction runtime. We also introduce a Binary Search Tree (BST)-based construction method, reducing time complexity and achieving comparable randomness but is much faster for some parametrizations. This is valuable for time-critical bioinformatics applications.

Additionally, we identify that the link function and comparator in the short-read mapper strobealign (Sahlin 2022) underperform in seed uniqueness compared to other methods. As a result, we modified strobealign to enhance accuracy. Although the modification does not improve the overall accuracy, an approach that selects the best alignment score per read from the modified and default versions of strobealign improves accuracy substantially. This finding can be used to further increase strobealign’s accuracy. In summary, our evaluation uncovers linking biases and offers guidance on operator selection for randstrobe implementations.

## 2 Materials and methods

### 2.1 Definitions

We use 0-indexed notation. We typically use *S* and *T* to denote strings, and we use the notation S[i:j], i < j to refer to a substring starting at position *i* and ending (and including) the character at position *j* in *S*. We let the |·| operator denote the length of strings. Here, our alphabet consists of the letters (or *nucleotides*) Σ={A,G,C,T}. We use h(x)→z, where *x* and *z* are integers to denote a hash function without specifying the underlying function. As for representation in memory, DNA strings shorter or equal to 32 nucleotides (nt) can be stored with 64-bit integers by encoding *A*, *C*, *G*, and *T* as 00, 01, 10, and 11, respectively. Other letters, such as *N* for “unknown” nucleotide, are ignored. For *k*-mers longer than 32 nt, we represent them as structs of (concatenated) 64-bit integers. We use the variable *x* to represent the integer value of the encoding. Finally, we use & for bitwise AND, ⊕ for bitwise XOR, ‖ for concatenation (e.g. concatenating two 64-bit integers into a 128-bit representation), and\% for the modulo operator. We also use B(x) to represent the function that returns the number of set bits in *x*.

### 2.2 An overview of constructing strobemers

A *k*-mer is a substring of *k* nucleotides in a biological sequence *S*. Consequently, a *k*-mer only needs the length of the substring, *k*, as a parameter to be specified. A strobemer is a set of linked *k*-mers. Specifically, a strobemer consist of n l-mers $l_{0},\ldots ,l_{n-1}$, denoted *strobes*, where the first strobe $l_{0}$ has a determined position *i* in *S*. Downstream strobe $l_{m}$, m∈[1,n − 1] is selected in an interval $S[i\;+\;w_{\min}+(m\;-\;1)w_{\max}:i\;+\;mw_{\max}]$ in *S*, and *linked* (appending the strobe to previous strobes) to the *m* previous strobes. Here, $w_{\min}$ and $w_{\max}$ specify the range of the sampling window. For example, strobe $l_{1}$ is sampled in $S[i\;+\;w_{\min}:i\;+\;w_{\max}]$ and linked to $l_{0}$.

Since we consider 64-bit integer representations of the strobes in this study, we will from now on refer to the strobes as $x_{0},x_{1},\ldots x_{n-1}$ and, when clear from context, we alternate *x* to mean either the strobe itself or its integer representation. This is also the reason we use the more general term *linking* instead of *appending* (strobes to the seed), as the linking method will vary with the strobe representation, as we discuss in detail in the next section.

The methods to select strobes differ (Sahlin 2021a). For example, Minstrobes have been used for long-read overlap detection (Firtina *et al.* 2023) and alternating strobe lengths have also been explored (Maier and Sahlin 2023). However, randstrobes were shown to be more sensitive for sequence matching than other methods using fixed strobe lengths (minstrobes and hybridstrobes) (Sahlin 2021a), and simpler to construct than alternating strobe lengths (altstrobes and multistrobes) (Maier and Sahlin 2023), and is so far most commonly implemented in practice (Sahlin 2022, Nip *et al.* 2023, Xu *et al.* 2023). Therefore, we will consider only the randstrobes method in this study. Randstrobes are parameterized by $\left(n,l,w_{\min},w_{\max}\right)$. The novelty compared to, e.g. *k*-mers and spaced *k*-mers is that strobemers allow flexibility in the strobes’ spacing and can produce matches between two sequences in a region with insertions or deletions.

### 2.3 Strobemer construction: constraints and objectives

Let $M_{w_{\min}}^{w_{\max}}(x_{i}|x_{i-1},\ldots ,x_{0})$, or simply *M* when context is clear, be a method to sample a strobe $x_{i}$ in a window given by its parametrization $\left(n,l,w_{\min},w_{\max}\right)$. We put the following constraints on *M*.

1. *M* selects $x_{i}$ based only on the sequence information of $x_{i-1},\ldots ,x_{0}$.
2. *M* is deterministic. That is, for two identical strings *S* and *T*, the same strobes are produced.

We want to find a method *M* such that

1. Maximize $H(M(x_{i}|x_{i-1},\ldots ,x_{0}))$, where *H* denotes the entropy. Intuitively, *M* should sample $x_{i}$ as uniform as possible within the window, regardless of previous strobes and the sequence in the window.
2. *M* constructs randstrobes as fast as possible.

The first constraint is essential to eliminate high-entropy but impractical solutions in sequence matching. For instance, using a (pseudo) random number generator (RNG) like rand() in C++ may seem to have good entropy. However, in scenarios involving similar strings *S* and *T*, where one has a deletion, the RNG is likely to generate different numbers upon encountering the deletion, making it unsuitable for string matching. Therefore, the method’s decision should solely rely on the underlying sequence.

The first objective, instead, involves conditional entropy, which is challenging to measure. Merely assessing entropy by the uniformity of sampling sites within a sequence window is insufficient. For instance, if a method prefers selecting a strobe if it is identical to the previous strobe, and the distance between two identical strobes happens to be uniformly distributed across a sequence, the method may falsely appear to have perfect entropy. It is also worth noting that achieving high entropy is easier in randomly generated sequences, but the focus here is on repetitive regions common in biological sequences, where achieving sampling uniformity is more challenging.

### 2.4 Constructing randstrobes

The process of creating randstrobes can be separated into four modular components:

1. Hashing the strobes;
2. Linking the strobes;
3. Comparing the strobes during linking;
4. Construction of the final seed hash value.

We discuss each of the components below and suggest different methods to perform them.

#### 2.4.1 Hashing strobes

Since each strobe is represented as a 64-bit integer using the binary encoding, the integers can further be hashed. The reason for hashing a strobe *x* as z=h(x) is that it can improve the pseudo-randomness. We evaluate the following hash functions for the strobes:

-
$h_{\text{NO}}(x)$ : The original 2-bit encoding of nucleotides is used without applying a hash function.
-
$h_{\text{TW}}(x)$ : *Thomas Wang hash* (http://web.archive.org/web/20071223173210/http://www.concentric.net/∼Ttwang/tech/inthash.htm), an invertible hash function used, e.g. in minimap2 (Li 2018).
-
$h_{\text{XX}}(x)$ : *xxHash* (https://xxhash.com/).
-
$h_{\text{WY}}(x)$ : *wyhash* (https://github.com/wangyi-fudan/wyhash).

Previously, $h_{\text{NO}}(x)$ was used in Sahlin (2021a) and $h_{\text{TW}}(x)$ was used (Sahlin 2022). This is the first study using $h_{\text{XX}}(x)$ and $h_{\text{WY}}(x)$ as hash functions to construct randstrobes. The hash functions xxHash and wyhash are general-purpose non-cryptographic pseudo-random hash functions that hash bytes into an integer range of size $2^{b}$ for some b>0 (here, b=64).

#### 2.4.2 Linking strobes

The second strobe $x_{1}$ is *linked* to the first strobe $x_{0}$ by selecting the candidate strobe $x_{1}^{\prime }$ in the window that minimizes or maximizes the link function ℓ. For example, in the first strobemers study (Sahlin 2021a), two link functions were used. The first was $\ell (x_{0},x_{1}^{\prime })=(x_{0}+x_{1}^{\prime })\;\mathrm{mod}\;p$, p∈Z [originally proposed in the preprint (Sahlin 2021b)]. The second one was $\ell (x_{0},x_{1}^{\prime })=(x_{0}+x_{1}^{\prime })\&q$, where *q* is a bitmask of 16 ones’ on the lowest significant bits and remaining 0s [proposed as faster alternative in the final publication (Sahlin 2021a)]. We call these functions $\ell_{\text{MOD}}$ and $\ell_{\text{AND}}$, respectively. Furthermore, two additional link functions were described in Sahlin (2022) and Xu *et al.* (2023) that we denote $\ell_{\text{BC}}$ and $\ell_{\text{XOR}}$. Here we propose three more alternatives: $\ell_{\text{XV}}$, $\ell_{\text{CC}}$, and $\ell_{\text{MAMD}}$. We provide formal definitions of all the link functions below.

-
$\ell_{\text{MOD}}(x_{0},x_{1})=(h(x_{0})\;+\;h(x_{1}))\;\mathrm{mod}\;p$ , p∈N (see Sahlin 2021a)
-
$\ell_{\text{AND}}(x_{0},x_{1})=(h(x_{0})\;+\;h(x_{1}))\&q,\;q\in N$ (see Sahlin 2021a)
-
$\ell_{\text{BC}}(x_{0},x_{1})=B(h(x_{0})\;⊕\;h(x_{1}))$ (see Sahlin 2022)
-
$\ell_{\text{XOR}}(x_{0},x_{1})=h(x_{0})\;⊕\;h(x_{1})$ (see Xu *et al.* 2023)
-
$\ell_{\text{XV}}(x_{0},x_{1})=h(x_{0}\;⊕\;x_{1})$ (proposed in this study)
-
$\ell_{\text{CC}}(x_{0},x_{1})=h(x_{0}||x_{1})$ [described in the pseudo code in Sahlin (2021a) but never studied]
-
$\ell_{\text{MAMD}}(x_{0},x_{1})=(h(x_{0})\;\mathrm{mod}\;p)+(h(x_{1})\;\mathrm{mod}\;p)\;\mathrm{mod}\;p$ , p∈N. Similar to $\ell_{\text{MOD}}$ but uses a BST (proposed in this study)

The $\ell_{\text{MAMD}}$ and $\ell_{\text{MOD}}$ are theoretically nearly identical (see Supplementary Section 1). However, $\ell_{\text{MAMD}}$ uses a BST to lower the time complexity. Consider a window of hash values. Roughly stated, the $\ell_{\text{MAMD}}$ link function only needs four operations as we are sweeping the window over the sequence; find minimum element (no modulo wrap-around), find the closest element to a specific value (modulo wrap-around), add incoming element, and remove outgoing element. These operations can all be performed in logarithmic time with a BST. The $\ell_{\text{MAMD}}$ link function is described in detail in Supplementary Section 1. We will discuss the computational complexity of all methods in Section 2.6. In this section, we only discussed linking the first two strobes. Linking additional strobes can be done recursively by applying the same link function between the previous resulting randstrobe hash value *b* with the next candidate downstream strobes $x_{m}$, m > 2 as $\ell (b,x_{m})$.

#### 2.4.3 Sampling comparator

The comparator function, here denoted c(·), specifies the criteria for which we select strobe $x_{1}$ among candidates $x_{1}^{\prime }$. To our knowledge, the only sampling comparator that has been proposed is $c_{\min}(x_{0},x_{1}^{\prime })=\mathrm{argmi}n_{x_{1}^{\prime }\in W}\ell_{\cdot }(x_{0},x_{1}^{\prime })$ (Sahlin 2021a, 2022, Xu *et al.* 2023), where *W* is the collection of strobes in the window defined by $w_{\min}$ and $w_{\max}$. In this study, we propose $c_{\max}(x_{0},x_{1}^{\prime })=\mathrm{argma}x_{x_{1}^{\prime }\in W}\ell (x_{0},x_{1})$. The comparator can influence the result for some hash and link constructions as we will see in our benchmark.

#### 2.4.4 The final seed hash value

We have so far discussed only how to select strobes. However, once the strobes have been decided, we need to represent the randstrobe with a *final hash value*. The final hash value is what should be indexed and queried, e.g. a seed-and-extend mapping framework. We denote the function to produce the final seed hash value as $f(x_{0},\ldots ,x_{n})$. We need the function *f* to be as uncorrelated with the link function as possible. If we would use the hash value that comes out of $\ell (x_{0},x_{1})$, with, e.g. $c_{\min}$, we are projecting hash values to the minimum value in each window. This leads to unnecessary hash collisions compared to a uniform hash function. Furthermore, as mentioned in Sahlin (2021a), it is important to avoid symmetric functions $f(x_{0},x_{1})=f(x_{1},x_{0})$ (e.g. $f(x_{0},x_{1})=x_{0}+x_{1}$) if distinguishing direction from, e.g. inversions is important [although a symmetric function is used to forward and reverse complements seeds in, e.g. read mapping (Sahlin 2022)]. Taking into consideration the above we use

$$\begin{array}{c} f(x_{0},x_{1},\ldots ,x_{n-1})=\{\begin{array}{cc} 2x_{0}-x_{1} & \text{if}\;n=2,\\ 2f(x_{0},x_{1},\ldots ,x_{n-2})-x_{n-1} & \text{if}\;n>2. \end{array} \end{array}$$

This formulation allows *f* not to have any apparent correlation with any of the benchmarked link functions, as we will see in Section 3.

### 2.5 Linking more than two strobes

Generally, to link $x_{m}$, to $x_{0},\ldots x_{m-1}$, m∈[2,n−1], we use $\ell (b,x_{m}^{\prime })$, where $x_{m}^{\prime }$ are the candidate strobes in the window, and *b* denotes a *base value* calculated from the previous *m* strobes. We set the *b* equal to the previous strobes’ final hash value, e.g. $b=f(x_{0},x_{1})$ and $\ell (b,x_{2}^{\prime })$ in the case of three strobes. This method can be applied recursively.

### 2.6 Time complexity

Before discussing computational complexity, we make the following classifications of our link functions:

- **Cheap computation**: This group includes $\ell_{\text{MOD}}$, $\ell_{\text{AND}}$, $\ell_{\text{BC}}$, $\ell_{\text{XOR}}$, and $\ell_{\text{MAMD}}$. We denote them as computationally cheap because the hashing and linking can be separated. That is, we only need to calculate hash values once for each strobe, and the link function can be applied after.
- **Expensive computation**: This group includes $\ell_{\text{CC}}$, and $\ell_{\text{XV}}$. For these methods, we need to evaluate the hash value for the combination of $x_{0}$ and all its candidate downstream strobes, for each new $x_{0}$.

The time complexity of constructing randstrobes from a string of length |*S*| varies with the link-function class. Let $t_{h}$ be the time complexity for the hash function, *n* the number of strobes, and $W=w_{\max}\;-\;w_{\min}+1$ be the window size. Then, $S\;-\;nw_{\max}\;-\;l\;+\;1$ the number of randstrobes constructed from *S*. We assume that the linking operators (i.e. +, &, ⊕, mod , ‖) can be performed in constant time, although the practical runtime varies among the operators with ⊕ being cheaper to perform while ‖ being relatively expensive.

Expensive computation methods perform (1+nW) hash calculations, and *nW* other operations (such as +, &, ⊕, mod , ‖), per randstrobe. So the total complexity is $O((|S|\;-\;nw_{\max}-l\;+\;1)((1\;+\;nW)t_{h}\;+\;nW))$. Cheap computation methods spend at most (|S| − l + 1) hash calculations and $\left(|S|\;-\;nw_{\max}\;-\;l\;+\;1)(nW\right)$ on other operations, in total. So the total complexity is $O((|S|\;-\;l\;+\;1)t_{h}\;+\;(|S|\;-\;nw_{\max}\;-l\;+\;1)(nW))$. If we assume that $|S|\gg nw_{\max}\;-\;l\;+\;1$ and $t_{h}=\Omega (1)$ (i.e. the complexity of $t_{h}$ is at least a constant), we can simplify the expression of the time complexity of expensive computation methods and cheap computation methods to $O(|S|nWt_{h})$, and $O(|S|t_{h}\;+\;|S|nW)$, respectively.

Lastly, the $\ell_{\text{MAMD}}$ link function is part of the cheap computation category. However, the time complexity is further reduced to $O(|S|t_{h}+|S|n\;\text{log}\;W)$ through the logarithmic time complexity of searching for elements (see Supplementary Section 1 for details). While the BST implementation increases the constant coefficient through the BST overhead, we will see that the speed-up is substantial for large windows. We have abstracted over the exact time complexity of the hash functions. The cheapest computation is $h_{NO}$ which only streams over the sequence without performing hashing. Some hash functions also support streaming (Mohamadi *et al.* 2016) and can lower $t_{h}$.

### 2.7 Evaluation metrics

There are different sampling biases that can arise as illustrated in Fig. 1. We were not able to find a singular metric that captured all of these biases, instead, we propose four suitable metrics that would capture cases B–E in Fig. 1. A desirable result is that the selection of the second (or any downstream) strobe is performed as uniformly in the window and as independently of the previous seed as possible. Several seed-based applications also require fast construction; therefore, we also benchmark construction runtime.

### 2.8 Notation for evaluation metrics

Let *N* be the total number of seeds constructed from a string *S*, and *M* the number of seeds with distinct final seed hash values in *S*. We let *i* and *j* be index variables over the set of randstrobes seeds sorted by their first strobe position. Since we here sample one randstrobe per position in *S*, the index variables are equivalent to the start position of the seed, and the *N* seeds can be ordered with respect to the start position on *S*. We let $s_{ik}$ refer to the *k*th strobe in seed *i* and $p_{ik}$ to its position in *S*.

### 2.9 E-hits

The E-hits metric was introduced in Sahlin (2022). It provides a number between 1 and |*S*|, which is the expected number of times a seed occurs in the reference. The E-hits metric was used as a measure for expected seed repetitiveness in *S* when sampling reads uniformly at random from a reference string *S*, assuming *S* is much larger than the span of the seed (Sahlin 2022). We restate the E-hits metric here for self-containment. Let i∈[1,M] be an index variable over the set of distinct seeds in *S* and N > M be the total number of seeds in *S* (multiset). Let $x_{i}$ denote the number of times seed *i* occurs in *S*. Let $q_{i}$ be the probability of producing seed *i* when selecting a seed randomly from the *N* seeds. The E-hits metric is then the expected value over seed hits *E*[*X*] computed as

(1) $$\begin{array}{c} E[X]=\sum_{i=1}^{M}q_{i}x_{i}=\sum_{i=1}^{M}\frac{x_{i}}{N}x_{i}=\frac{1}{N}\sum_{i=1}^{m}x_{i}^{2}. \end{array}$$

In this study, seeds are represented as hash values. The above formula is equivalent if we replace the notion of a seed with the hash value representation of a seed. In this case, E-hits measure the expected number of identical hash values, which includes both repetitive seeds and non-desired hash collisions. We will measure the E-hits for the final seed hash values produced with *f*, and denote this quantity $E_{f}$. This is the same use of E-hits as in Sahlin (2022).

### 2.10 E-hits of inter-strobe distance and strobe position

The idea and formulation of E-hits can be used to measure the repetitiveness of other quantities. To measure strobe-distance clumping (bias B) and periodicity clumping (bias D) in Fig 1, we look at the distribution of inter-strobe distances within a randstrobe. Let $d_{jk}$ be the distance between the first strobe and the *k*th strobe in seed *j*. We let $x_{i}$ in Equation (1) be the number of times we observe distance $d_{jk}$. Equation (1) then measures the expected number of times we observe the distance $d_{jk}$ when randomly drawing a seed from *S*. We denote this quantity as $E_{d}$ and omit index variable *k* when it is clear from the context.

We measure second-strobe clumping (bias C) by computing the repetitiveness of the position of *k*th strobes in *S*. Let $x_{i}$ in Equation (1) represent the number of times we observe the *k*th strobe selected at position *p* in *S*. Then, the E-hits formula measures the expected number of times position *p* was sampled as the *k*th strobe when drawing a seed uniformly at random from *S*. We denote this quantity as $E_{p}$ (omitting index variable *k* when clear from context).

### 2.11 The conflict metric

To study complex dependencies (termed other clumping; Case E) as depicted in Fig. 1, we introduce the *conflict metric*, which aims to measure the size of the overlaps of strobes from a set of neighboring randstrobes with start positions in [*i*, *j*], i < j. An overlap higher than what is expected under random sampling indicates selection bias. Let $o(i,j,k)=\max(0,l\;-\;|p_{jk}\;-\;p_{ik}|)$ measuring the number of overlapping positions of the *k*th strobe between two randstrobes *i* and *j*. Then $\sum_{k=0}^{n-1}o(i,j,k)$ is the total number of overlapping positions between two randstrobes. The conflict metric for randstrobe *i* is then defined as

$$C_{i}=\underset{j\in [i+1,\min(N,i+m)]}{\max}\sum_{k=0}^{n-1}o(i,j,k).$$

In other words, $C_{i}$ is the largest observed overlap with any of the *m* consecutive downstream randstrobe seeds. We let the conflict metric (*C*) be the value of $C_{i}$ averaged over all seeds in *S*. The above formula does not take into account that strobes of different orders (*k*) between neighboring randstrobes might overlap. However, even if this is possible for some values of $w_{\min}$, it does not originate from the bias that we want to measure, and can therefore be omitted.

## 3 Results

We evaluated all compatible combinations of ℓ,c, and *h*. Some combinations, such as $h_{TW}$ with $\ell_{CC}$, are incompatible with strobes larger than 16 nucleotides (32 bits) because $h_{TW}$ is designed for 64-bit integers. We use a simulated highly repetitive sequence (SIM), a set of 20 *Escherichia coli* genomes (E20), and the CHM13 human chromosome Y from the T2T assembly (Nurk *et al.* 2022) (ChrY) to evaluate pseudo randomness for randstrobes with n=2. For runtime experiments, we used a simulated string of length 15 million. We also evaluated randstrobes n=3 on the SIM dataset. Details of the experiment design and rationale are found in the Supplementary Section 2.

### 3.1 Pseudo-randomness

As for pseudo-randomness, we observed similar trends for the methods across the SIM, E20 and ChrY datasets. We also observed that the three hash functions ($h_{WY},h_{TW},h_{XX}$) had very similar results, we therefore focus on presenting the data for the SIM dataset using only $h_{WY}$ compared to not hashing ($h_{NO}$) here. Results with all hash functions for SIM, E20, and ChrY are found in Supplementary Materials. Our benchmark highlights the following takeaways.

**Figure 2.**
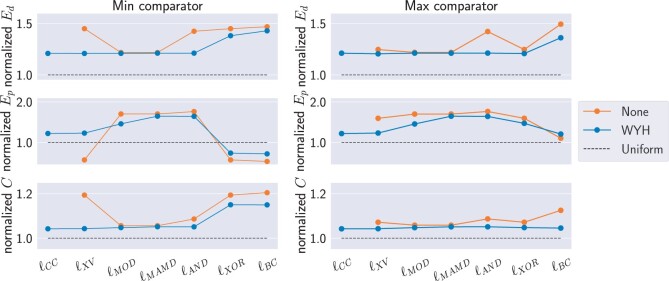
Results for metrics $E_{d}$ (upper panels), $E_{p}$ (middle panels), and *C* (lower panels) for randstrobes with parameter settings $\left(n=2,l=20,w_{\min}=21,w_{\max}=100\right)$ for the repetitive sequence dataset. The *x*-axis shows the different linking methods, and the min and max comparators are shown in left and right panels, respectively. We have normalized the values with a near ideal result produced by simulating strobes uniformly at random in the window with rand(). Therefore, a value of 1.0 indicates best possible outcome (indicated by black dashed line).

**Hashing strobes:** Always use a hash function to hash the strobes before linking (applicable to all link functions except $\ell_{CC}$), otherwise most link functions will be subject to some form of severe bias (Fig. 2 and Supplementary Figs S1–S3).

**Link function:** The two expensive methods ($\ell_{XV},\ell_{CC}$) achieve the best pseudo-randomness (Fig. 2 and Supplementary Figs S1–S3)). As for the computationally cheap methods, different methods have different bias (Table 1).

**Table 1.** Overview of link functions and comparator functions based on the results from our experiments.^a^

Category	ℓ	*c*	Introduced	Bias	Speed	Uniqueness	Comment (strength/weakness).
Expensive	$\ell_{CC}$	Any	This study^b^	—	Slow	High	Slow but supreme randomness.
	$\ell_{XV}$	Any	This study	—	Slow	High	Slow but supreme randomness.
	$\ell_{\mathrm{XOR}}$	$c_{\min}$	(Xu *et al.* 2023)	$E_{p},E_{d},E_{c}$	Fast	Low	XOR with $c_{\min}$ collapse similar regions leading to
							repetitiveness. Application determines if desired.
		$c_{\max}$	This study	$E_{p}$	Fast	High	Fastest method with good randomness.
Cheap	$\ell_{\mathrm{MAMD}}$	Any	This study	$E_{p}$	Fast^c^	High	Only method to scale for very large windows (>1000).
	$\ell_{\mathrm{AND}}$	Any	(Sahlin 2021a)	$E_{p}$	Fast	High	Fast but higher $E_{p}$ than $\ell_{\mathrm{XOR}}$.
	$\ell_{\mathrm{MOD}}$	Any	(Sahlin 2021b)	$E_{p}$	Medium	High	Slower than $\ell_{\mathrm{XOR}}$ but not sensitive to the comparator.
	$\ell_{BC}$	$c_{\min}$	(Sahlin 2022)	$E_{p},E_{d},E_{c}$	Slow	High	Designed to be biased. sampling at the beginning of
		$c_{\max}$	This study	$E_{p},E_{d}$	Slow	High	The window more often. As slow as expensive methods.

a Results are described under the assumption that a hash function is used to hash the strobes (applicable to all link functions except $\ell_{CC}$).

b Mentioned in Sahlin (2021a) but neither used nor studied.

c Too much overhead to be used for small windows.

**Comparator:** Comparator choice is only important for some link functions. Cheap computation XOR-based methods $\ell_{\mathrm{XOR}}$ and $\ell_{BC}$ exhibit high bias with the $c_{\min}$ comparator. This is because the $c_{\min}$ comparator will select a candidate strobe to be identical to the previous strobe if present in the window (XOR value of 0) while $c_{\max}$ will have the opposite behavior. Since our repeats in the SIM dataset have reoccurring distances between them (which also happens in biological sequences), it causes distance clumping (bias B) and negative positional clumping (bias C).

### 3.2 Seed repetitiveness

**Figure 3.**
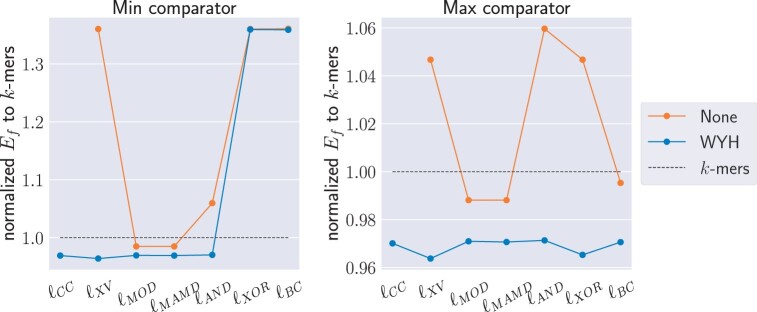
Normalized E-hits of seed hash values for various to construct randstrobes with parameters $\left(n=2,l=20,w_{\min}=21,w_{\max}=100\right)$ compared to *k*-mers of size 40. Lower value is better.

Seed repetitiveness in the reference is crucial for applications such as read mapping (Sahlin 2022, Ekim *et al.* 2023, Maier and Sahlin 2023, Shaw and Yu 2023). We use *k*-mers of length 40 nt, corresponding to the same number of sampled positions in the randstrobes, as a reference method in this benchmark. The *k*-mers are stored as two strobes with the same final function as the randstrobes, $f(x_{0},x_{1})=2x_{0}-x_{1}$. We first verified that using our final hash function *f* for seed representation resulted in minimal hash collisions (Supplementary Fig. S4). Since hash collisions were not significant, we computed the E-hits of the final seed hash value ($E_{f}$), for all methods. As with randomness, it is important to use a hash function before linking strobes (Fig. 3 and Supplementary Figs S1–S3). Additionally, we observed that randstrobes generally have lower $E_{f}$ than k-mers for most hash and link functions, but repetitiveness can increase with specific combinations (Fig. 3).

### 3.3 Runtime performance

**Figure 4.**
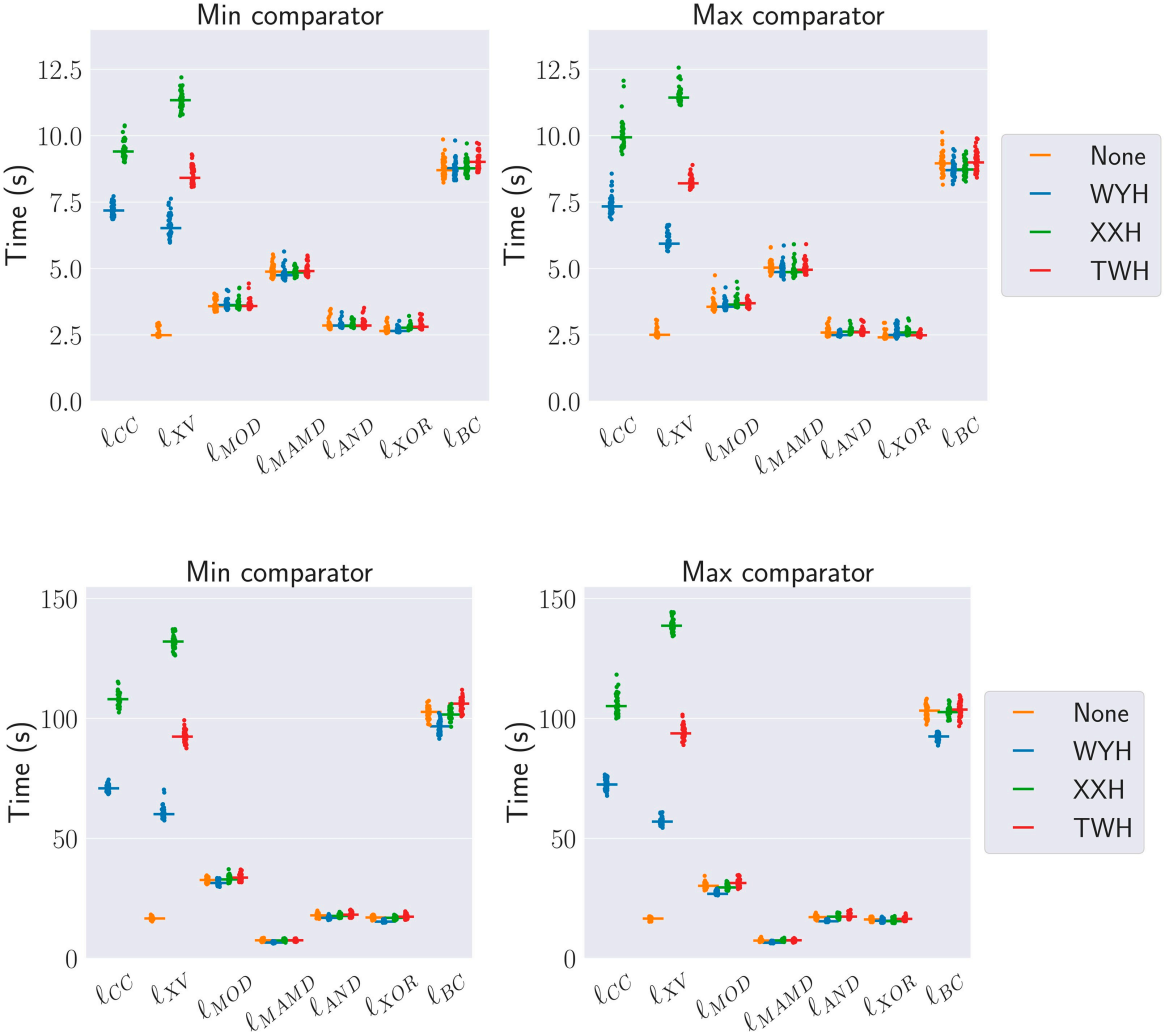
Runtime (seconds) on 45 instances for each combination on a 15 million nt simulated string. Each combination generates randstrobes with n=2, l=20, $w_{\min}=21$, and $w_{\max}=100$ (upper panel) and $w_{\max}=1000$ (lower panel).

Figure 4 shows the construction time for window sizes using $w_{\max}=100$ and $w_{\max}=1000$, respectively. Expensive computation methods ($\ell_{CC}$ and $\ell_{XV}$) are performing a factor of *nW* more hash computations. However, they are only about 2.5–4 times slower than the average cheap computation methods when using $h_{WY}$ as hash function (Fig. 4). One explanation could be cache efficiency. We also observe that the $\ell_{BC}$ and $\ell_{\mathrm{MOD}}$ are substantially slower than other methods in the cheap-computation class. Finally, when constructing randstrobes with large windows, $\ell_{\mathrm{MAMD}}$ is much faster than other methods (Fig. 4, lower panels). This is due to the BST implementation instead of a linear search across each window. However, due to its special updating technique utilizing arithmetic properties of the modulo operator, the method can only be used with the modulo link function. As for the hash functions, $h_{WY}$ performs better than $h_{XX}$ and $h_{TW}$ on our data for the expensive computation class, where strobes are represented by a struct of two 64-bit integer strobes.

### 3.4 Randstrobes in large windows

**Figure 5.**
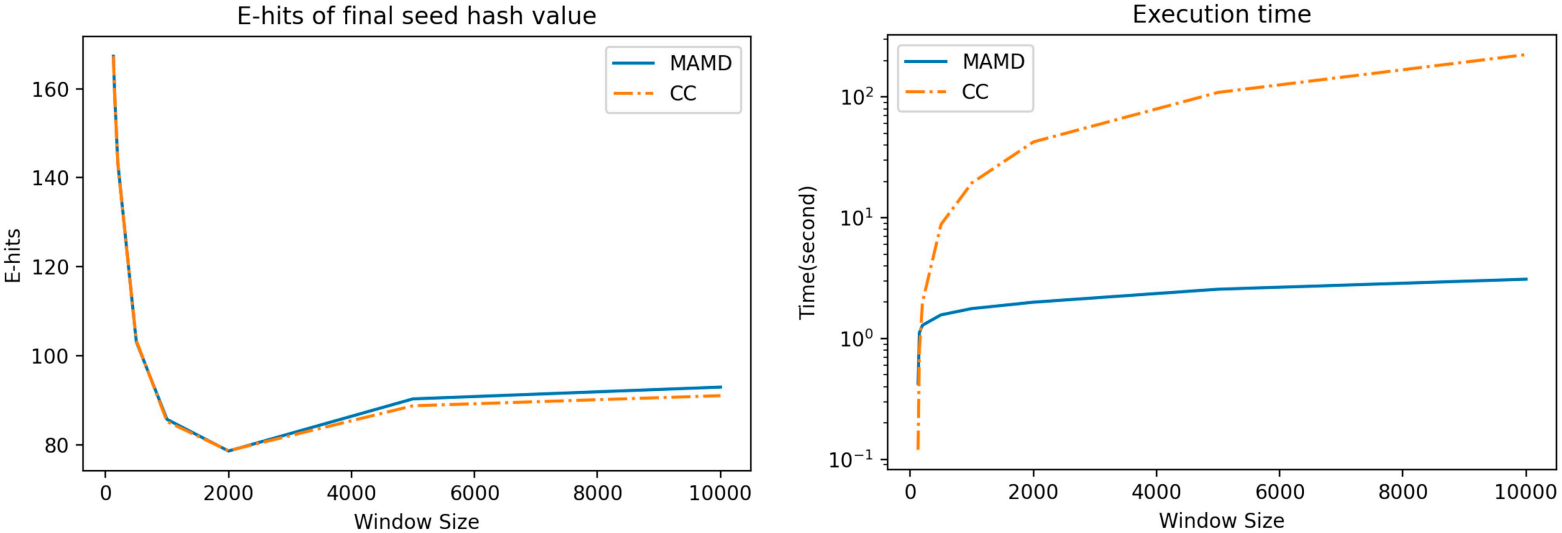
A comparison between $\ell_{\mathrm{MAMD}}$ and $\ell_{CC}$ with parameters $\left(n=2,l=128,w_{\min}=129,w_{\max}=x\right)$, where *x* is plotted on the *x*-axis. Left panel shows E-hits on Chromosome Y from the CHM13 human assembly (Nurk *et al*. 2022). The right panel shows median runtime out of 10 runs on an *E.coli* genome of 5.5 million nucleotides.

The $\ell_{\mathrm{MAMD}}$ link function enables efficient construction of randstrobes in large windows. We were interested in the uniqueness of seeds that $\ell_{\mathrm{MAMD}}$ produced compared to one of the best-performing methods $\ell_{CC}$ (using $c_{\max}$). We used p=100,001 in the previous analysis. For this analysis, we set p=19,019,684,767,739,993. The value of *p* needs to be significantly larger than the window size but smaller than the maximum hash value to guarantee high pseudo-randomness. To our knowledge, the value of *p* has no specific influence beyond that. We investigated the expected uniqueness (E-Hits) of the seeds computed across chromosome Y of the CHM13 assembly (Fig. 5, left panel). In the figure, a window size of 0 corresponds to *k*-mers of size 256. We make two key observations about the uniqueness of seeds. First, we note that there is no substantial difference between the two link functions on chromosome Y from the CHM13 assembly, including telomere regions and many repetitive multigene families. Second, we observe that the E-hits function is not linearly decreasing, which we initially expected. Minimum repetitiveness occurs at $w_{\max}=2,000$ instead of the largest evaluated window at $w_{\max}=10,000$. This is likely explained by the observation that, beyond a certain window size, the more likely it is that the same pair of strobes is found and linked. We also looked at how the runtime scaled with window size. Figure 5 (right panel) shows the median runtime from 10 runs on the *E.coli* genome of 5.5 million nucleotides. Our BST implementation greatly outperforms $\ell_{CC}$.

### 3.5 Implementing $c_{\max}$ in strobealign

Strobealign is a read mapper that use randstrobes created from syncmers (Edgar 2021) using $c_{\min}$ together with $\ell_{BC}$, which we observed were particularly bad in terms of seed uniqueness and randomness (Figs 2 and 3). Guided by our benchmark, we wanted to investigate whether $c_{\max}$ would result in better mapping results. The experiment is described in detail in Supplementary Section 4. We did not observe a direct improvement in strobealign’s accuracy when run with $c_{\max}$ compared to the default version that uses $c_{\min}$ (Supplementary Tables S1 and S2). However, we observed a large improvement in accuracy for the shorter read lengths when combining the results of the two runs of strobealign (details in Supplementary Section 4).

## 4 Discussion and conclusions

Constructing randstrobes involves four modular operations: computing individual strobe hash values (hash function), determining hash values for linked strobes (link function), selecting the final randstrobe from multiple candidates using a comparator function, and computing the hash value for the chosen randstrobe. The initial three operations (hash, link, and comparator functions) yield diverse results based on the combination of functions used. Our study introduced and benchmarked both novel and previously used hash, link, and comparator methods for randstrobe construction, accompanied by metrics to evaluate method biases. Our benchmark revealed biases in existing techniques and can offer general guidance for which methods to use when utilizing randstrobes as sequence comparison seeds. From our evaluation, we conclude the following.

- **Hashing:** Always hash the strobes before linking with a computationally cheap link method. It does not result in a large overhead in construction time (Fig. 4) while being beneficial for pseudo-randomness (Figs 2 and 3). The hash functions have roughly the same pseudo-randomness performance, but the $h_{WY}$ function had the best runtime. A downside with hashing compared to the 2-bit encoding is that nucleotide level information of the seed is lost. This should be factored into the decision for the application at hand.
- **Linking:** In short, we believe $\ell_{CC}$ or $\ell_{XV}$ should be used when highest pseudo-randomness is desired, $\ell_{\mathrm{XOR}}$ (with $c_{\max}$) should be used when speed is important, and $\ell_{\mathrm{MAMD}}$ for use cases with very large windows (Table 1). We do not see any benefit with using $\ell_{\mathrm{AND}}$ and $\ell_{\mathrm{MOD}}$ over $\ell_{\mathrm{XOR}}$. Finally, $\ell_{BC}$ is a special function designed for when biased sampling is desired, as in Sahlin (2022).
- **Comparator:** The comparator matters for some link functions (Table 1). For example, an XOR-based link-function projects identical hash values to 0. Therefore, a min comparator will select identical strobes if present, while a max comparator will be inclined to select differing strobes. Consequently, in repetitive regions with occasional variations (e.g. SIM dataset) where the window is larger than the repeat length, the min comparator will tend to collapse seeds while a max comparator has the opposite behavior. This however implies that in such regions, the max comparator will be less robust to sequencing errors in reads. These two effects pull in different directions when it comes to read mapping. We observed no substantial difference between them in strobealign (Supplementary Tables S1 and S2) but combining their results led to large improvement for shorter reads (Supplementary Tables S1 and S2).
- **Final seed hash value function:** Choose a final seed hash value function that is uncorrelated to the link function to avoid hash collisions. For example, we used $2x_{0}-x_{1}$ for two strobes that did not show any apparent correlation with the link functions we benchmarked (Supplementary Fig. S4).

## 5 Future work

Efficiently applying hash and link functions can benefit cheap computation methods. A rolling hash function, like ntHash (Mohamadi *et al.* 2016), can enhance hash computation in these methods. This optimization proves valuable when hashing is relatively more expensive than linking, particularly for larger window sizes. Additionally, a link function $\ell_{\text{MAMD}}$ was designed using arithmetic reasoning to reduce construction time complexity. Further investigation is needed to determine if the rolling hash approach allows for arithmetic operations permitting efficient linking methods.

We observed improved accuracy when combining results from min and max comparators in strobealign. Our proof-of-concept approach involved running strobealign twice and post-processing the alignments, resulting in slightly more than twice the runtime compared to a single run. To mitigate an increase in runtime, integrating seeds from both comparators into strobealign is a solution. This increases memory usage but may not affect runtime since costly rescue-alignment calls may lowered due to fewer regions without seed matches.

## Supplementary Material

btae187_Supplementary_Data

## Data Availability

The human Y chromosome from the CHM13 genome was downloaded from https://github.com/marbl/CHM13?tab=readme-ov-file#downloads and the 20 E. coli strains used in the E20 benchmark is found in supplementary materials and can be downloaded from RefSeq (https://www.ncbi.nlm.nih.gov/refseq/). The code for generating the simulated dataset is available at https://github.com/Moein-Karami/RandStrobes.

## References

[btae187-B1] Alser M , Rotman J, Deshpande D et al Technology dictates algorithms: recent developments in read alignment. Genome Biol 2021;22:249. 34446078 10.1186/s13059-021-02443-7 PMC8390189

[btae187-B2] Chin C-S , Khalak A. Human genome assembly in 100 minutes. bioRxiv, 10.1101/705616, 2019, preprint: not peer reviewed.

[btae187-B3] Edgar R. Syncmers are more sensitive than minimizers for selecting conserved k-mers in biological sequences. PeerJ 2021;9:e10805. 10.7717/peerj.10805. 33604186 PMC7869670

[btae187-B4] Ekim B , Berger B, Chikhi R. Minimizer-space de Bruijn graphs: whole-genome assembly of long reads in minutes on a personal computer. Cell Syst 2021;12:958–68.e6. 34525345 10.1016/j.cels.2021.08.009 PMC8562525

[btae187-B5] Ekim B , Sahlin K, Medvedev P et al Efficient mapping of accurate long reads in minimizer space with mapquik. Genome Res 2023;33:1188–97. 10.1101/gr.277679.123. 37399256 PMC10538364

[btae187-B6] Fan J , Khan J, Singh NP et al Fulgor: a fast and compact k-mer index for large-scale matching and color queries. Algorithms Mol Biol 2024;19:1–21. 38254124 10.1186/s13015-024-00251-9 PMC10810250

[btae187-B7] Firtina C , Park J, Alser M et al Blend: a fast, memory-efficient and accurate mechanism to find fuzzy seed matches in genome analysis. NAR Genom Bioinform 2023;5:lqad004. 36685727 10.1093/nargab/lqad004 PMC9853099

[btae187-B8] Li H. Minimap2: pairwise alignment for nucleotide sequences. Bioinformatics 2018;34:3094–100. 29750242 10.1093/bioinformatics/bty191 PMC6137996

[btae187-B9] Ma B , Tromp J, Li M. PatternHunter: faster and more sensitive homology search. Bioinformatics 2002;18:440–5. 10.1093/bioinformatics/18.3.440. 11934743

[btae187-B10] Maier BD , Sahlin K. Entropy predicts sensitivity of pseudo-random seeds. Genome Res 2023;33:1162–74. 10.1101/gr.277645.123. 37217253 PMC10538493

[btae187-B11] Marchet C , Boucher C, Puglisi SJ et al Data structures based on k-mers for querying large collections of sequencing data sets. Genome Res 2021;31:1–12. 10.1101/gr.260604.119. 33328168 PMC7849385

[btae187-B12] Mohamadi H , Chu J, Vandervalk BP et al ntHash: recursive nucleotide hashing. Bioinformatics 2016;32:3492–4. 27423894 10.1093/bioinformatics/btw397 PMC5181554

[btae187-B13] Nip KM , Hafezqorani S, Gagalova KK et al Reference-free assembly of long-read transcriptome sequencing data with rna-bloom2. Nat Commun 2023;14:2940. 37217540 10.1038/s41467-023-38553-y PMC10202958

[btae187-B14] Nurk S , Koren S, Rhie A, et al The complete sequence of a human genome. Science 2022;376:44–53. 35357919 10.1126/science.abj6987 PMC9186530

[btae187-B15] Roberts M , Hayes W, Hunt BR et al Reducing storage requirements for biological sequence comparison. Bioinformatics 2004;20:3363–9. 10.1093/bioinformatics/bth408. 15256412

[btae187-B16] Sahlin K. Effective sequence similarity detection with strobemers. Genome Res2021a;31:2080–94. 10.1101/gr.275648.121. 34667119 PMC8559714

[btae187-B17] Sahlin K. Strobemers: an alternative to k-mers for sequence comparison. bioRxiv, 2021b, preprint: not peer reviewed.

[btae187-B18] Sahlin K. Strobealign: flexible seed size enables ultra-fast and accurate read alignment. Genome Biol 2022;23:260. 36522758 10.1186/s13059-022-02831-7 PMC9753264

[btae187-B19] Sahlin K , Medvedev P. Error correction enables use of Oxford Nanopore technology for reference-free transcriptome analysis. Nat Commun 2021;12:2. 10.1038/s41467-020-20340-8. 33397972 PMC7782715

[btae187-B20] Sahlin K , Baudeau T, Cazaux B et al A survey of mapping algorithms in the long-reads era. Genome Biol 2023;24:133. 37264447 10.1186/s13059-023-02972-3 PMC10236595

[btae187-B21] Shaw J , Yu YW. Proving sequence aligners can guarantee accuracy in almost o(m log n) time through an average-case analysis of the seed-chain-extend heuristic. Genome Res 2023;33:1175–87. 10.1101/gr.277637.122. 36990779 PMC10538486

[btae187-B22] Xu M , Guo L, Qi Y et al Symbiont-screener: a reference-free tool to separate host sequences from symbionts for error-prone long reads. Front Mar Sci 2023;10. 10.3389/fmars.2023.1087447.

