# Supplementary material for: Designing efficient randstrobes for sequence similarity analyses
Source: Bioinformatics. 2024 Apr 5;40(4):btae187. doi: 10.1093/bioinformatics/btae187 (PMC11034988; doi:10.1093/bioinformatics/btae187)
Supplement: btae187_Supplementary_Data [file btae187_supplementary_data.pdf]

Supplementary data for: Designing efficient randstrobes for sequence similarity analyses

February 23, 2024

1 ℓ_{MAMD} implementation

Link functions ℓ_{MOD} and ℓ_{MAMD} use the same modulo operation. However, the ℓ_{MOD} can overflow the integer limit $(2^{64}-1)$, while ℓ_{MAMD} can not if $p < 2^{63}-1$. The bigger difference lies in their implementation, and thus, computational complexity. The ℓ_{MAMD} link function reduces the time complexity of the construction through the use of a Binary Search Tree (BST). The ℓ_{MAMD} method is implemented as follows. Consider the min comparator and a BST B containing all the possible candidates x_1' for the second strobe x_1 by storing the hash of each strobe modulo p. We want to choose $x_1 = \text{argmin}_{x_1'}(x_0 + x_1') \mod p$. First note that we have $0 \leq x_0 + x_1' < 2p$ for all possible strobes (x_1') in the window, because we store all the hash values modulo p. There are two possibilities for the best value x_1' that we discuss separately.

- If $x_0 + x_1' < p$, the best possible candidate is the smallest value in the window. This is because for any other value x_1'' that is greater than x_1' and satisfies $x_0 + x_1'' < p$, we have $x_1'' > x_1' \implies x_0 + x_1'' \mod p > x_0 + x_1' \mod p$.
- If $p \leq x_0 + x_1' < 2p$, the best possible candidate is the smallest value that is equal to or greater than $p - x_0$ in the window. This is because for any other value x_1'' that is greater than x_1', we have $x_0 + x_1'' > p$ and $x_1'' > x_1' \implies x_0 + x_1'' \mod p > x_0 + x_1' \mod p$. For any other value x_1''' that is smaller than x_1', we have $x_1''' + x_0 < p$, which falls under the previous situation.

Therefore, the only two candidates for x_1' are the minimum value in B and the smallest value that is equal to or greater than $p - x_0$. We can compare the two values and select the best one. These values can both be found in $O(\log(W_{\max} - W_{\min}))$ time, where $W_{\max}$ and $W_{\min}$ are the boundaries of the window. Finding the minimum element in a BST is a standard operation. For the second case, in the BST implementation we use (std::set in C++), we can find the greatest value $y \leq p - x_0$. We can then find the next element in $O(1)$ which implies the smallest value that is greater than $p - x_0$.

To create the next randstrobe, the window swaps the value corresponding to the leftmost value in the window with an incoming value (rightmost value in the new window). Removing and adding values are also $O(\log(W_{\max} - W_{\min}))$ operations in a BST. If a max comparator is used, we have an analogous case.

2 Experiment setup

It is easy to produce randstrobes with high entropy if the underlying sequence in $[w_{min}, w_{max}]$ has high entropy (e.g., randomly generating letters in {A,C,G,T}). Therefore, we are interested in evaluating pseudo randomness in repetitive regions, common in biological sequences. We use a simulated highly repetitive sequence (denoted SIM), 20 E. coli genomes (denoted E20), and the chromosome Y from the CHM13 human assembly [5] including telomere regions (denoted ChrY).

2.1 SIM

We simulated a repetitive sequence S as follows. A sequence T consisting of 25 nucleotides A, G, C, and T was randomly generated and appended to S. We then simulated a new copy of T' from T by mutating each

position in T with a probability of $p = 0.02$, where the mutation could either be a substitution, insertion, or deletion with equal probabilities. T' was then appended to S and used as the new template to simulate the next copy T''. This recursive procedure was repeated 40,000 times. If the length of any template copy decreased to below 15 nucleotides, we only considered substitutions and insertions for those templates. This process resulted in a string of approximately 1,5 million nucleotides.

2.2 E20

We downloaded and used the following 20 *E. coli* chromosome sequences (*i.e.*, removing plasmids) from RefSeq:

```
>NZ_CP116919.1_Escherichia_coli_strain_MLI102_chromosome
>NZ_CP117008.1_Escherichia_coli_strain_MLI109_chromosome
>NZ_CP117013.1_Escherichia_coli_strain_MLI114_chromosome
>NZ_CP117016.1_Escherichia_coli_strain_MLI121_chromosome
>NZ_CP123240.1_Escherichia_coli_strain_YZLc1-3_chromosome
>NZ_AP027159.1_Escherichia_coli_strain_2313_chromosome
>NZ_AP027162.1_Escherichia_coli_strain_EH031_chromosome
>NZ_AP027165.1_Escherichia_coli_strain_H19_chromosome
>NZ_AP027170.1_Escherichia_coli_strain_20.1_chromosome
>NZ_AP027176.1_Escherichia_coli_strain_EH2252_chromosome
>NZ_AP027181.1_Escherichia_coli_strain_98E11_chromosome
>NZ_AP027185.1_Escherichia_coli_strain_NIID080884_chromosome
>NZ_AP027188.1_Escherichia_coli_strain_PV0838_chromosome
>NZ_AP027191.1_Escherichia_coli_strain_10153_chromosome
>NZ_AP027197.1_Escherichia_coli_strain_02E060_chromosome
>NZ_CP116067.1_Escherichia_coli_strain_DETEC-S792_chromosome
>NZ_CP116071.1_Escherichia_coli_strain_DETEC-S589_chromosome
>NZ_CP116145.1_Escherichia_coli_strain_DETEC-P622_chromosome
>NZ_CP116035.1_Escherichia_coli_strain_FAH_chromosome
>NZ_CP093548.1_Escherichia_coli_strain_YL03_chromosome
```

2.3 ChrY

The Y chromosome from the T2T assembly was obtained from `https://github.com/marbl/CHM13?tab=readme-ov-file#downloads`.

2.4 Parameters and evaluation

We used randstrobe parameter value of $n = 2, l = 20, w_{min} = 21, w_{max} = 100$. In addition, we also report the results for three strobes ($n = 3$) in Suppl. Section 3. Since the metrics we use could be difficult to interpret in a vacuum, we have, when applicable, also included suitable reference values. These reference values could either be k-mers with size $k = nl$, or a fully random method, denoted *uniform*, that produces randstrobes by uniformly at random selecting a position in the sampling window $[w_{min}, w_{max}]$ (using `rand()` in C++). We remark that this method produces different randstrobes from the same sequence. Thus, we cannot use uniform randstrobes for anything other than providing best-case reference values for other methods. We use $p = 100,001$ for ℓ_{MAMD} and ℓ_{MOD} in our experiments.

To evaluate runtime, we use a simulated 15 million nucleotides string with nucleotides chosen uniformly at random. We report the median runtime as well as the individual runtimes on 45 replicate runs. We consider the difference between the starting and the finishing time of creating and storing randstrobes in a vector as the execution time. The experiments were run on an Linux server with an Intel Xeon E3-12xx v2 (Ivy Bridge, IBRS) CPU, and compiled with gcc with flag `-O3`. For the runtime, we evaluated randstrobes parametrized as ($n = 2, l = 20, w_{min} = 21, w_{max} = 100$) and ($n = 2, l = 20, w_{min} = 21, w_{max} = 1000$) since the window size affects runtime.

3 Results for randstrobes with three strobes

We also investigated constructing randstrobes with $(n = 3, l = 20, w_{min} = 21, w_{max} = 100)$. In general, we observed similar behavior in terms of pseudo-randomness as for the construction of randstrobes with two strobes (Fig. S5). This is expected due to the recursive nature of the construction. That is, when selecting the m-th strobe, we perform the selection based on a base value constructed from previous strobes (described in section 2.3). This process repeats recursively. When $m = 2$, the base value is simply the hash value of the first strobe.

As for the uniqueness of seeds, as demonstrated in [6], randstrobes with three strobes are relatively more unique than k-mers with the same number of sampled bases (Fig. S5, $k = 60$) . This is due to the increase in range of the seed, thus, the increased number of options available for selecting strobes. But some combinations (e.g., no hashing or based on the XOR operator; Fig. S5) can reduce the uniqueness.

Strobemers with $n > 3$ show no substantial gain in the context of sequence matching at the cost of additional runtime [4] (although they have been modified and used for specific scenarios [2]). Also, the relative performance can be extrapolated from the $n = 2$ and $n = 3$ cases, since the construction is recursive, therefore, we omit them in this study.

4 Implementing c_{max} in strobealign

We observed in our benchmark (Fig. 2) that c_{min} together with ℓ_{BC} were particularly bad in terms of seed uniqueness and randomness (Fig. 2 and 3). Strobealign [7] is a short-read mapper that uses ℓ_{BC} together with the c_{min}. Guided by our benchmark, we wanted to investigate whether c_{max} would result in better mapping results. First, strobealign adds other modifications to the strobemer constructions, such as thinning out the k-mers by using syncmers [1], masking the majority of bits before applying ℓ_{BC}, and applying customized window sizes (w_{min} and w_{max}) based on thinning rate and read length. Such modifications may make the observations from our analysis less effective or even inapplicable to the seeding within strobealign.

Nevertheless, we evaluated the accuracy of strobealign (v0.11.0) when mapping reads to the drosophila, CHM13, maize, and rye genomes used in [7] for read lengths 50, 75, 100, 150, 200, 250, 300, 500 by simulating one million read pairs (reads if single-end experiment) for each instance. While we did not observe a direct improvement in accuracy only when comparing the accuracy results between the two versions for neither paired-end (Table S1) nor single-end reads (Table S2), we observed a large improvement in accuracy when combining the results of the two runs of strobealign. In the case of the paired-end reads (Table S1), the combined results were obtained as follows. We pick the alignment from c_{max} if the read pair was properly paired with c_{max} but not with c_{min} or the sum of alignment scores for c_{max} was higher than for c_{min} where unmapped reads count as having a score of 0. Otherwise, we picked the result from c_{min}. For the single-end reads, the combined results were obtained by selecting the best alignment per read (decided by alignment score) when comparing the SAM files, where unmapped reads count as having a score of 0.

We observed that both in the paired-end and single-end experiments, the shorter read lengths benefited the most from combining the results (seen from the percentage point difference in Table S1 and S2. The generally most accurate aligner in the benchmark of strobealign [7] was BWA-MEM [3]. We included the percentage point difference to BWA-MEM in Table S1 and S2, where a negative result indicates that the accuracy from the combined experiment was more accurate than BWA-MEM. While strobealign is more accurate when aligning reads to drosophila for most read lengths, BWA-MEM is more accurate on the larger genomes CHM13, rye, and maize. However, by comparing the diff column to the combined diff to BWA column, the percentage point difference to BWA-MEM is decreased by half or more for many of the datasets, when combined results are used.

For the single-end experiment, we also generated a combined version where we only used the c_{max} result if a read was unmapped with the default strobealign (i.e., using c_{min}). This version also generated a relatively large increase in accuracy, particularly for the shorter reads (Table S3). This suggests that expensive alignment rescue steps (both in the seeding step and in the alignment step) may be avoided for the shorter reads by having more matching seeds.

In addition, we observed no apparent difference in the number of mapped reads, memory usage, and runtime between the two versions of strobealign. Our results suggest a mapping strategy where the c_{min} and c_{max} comparators could be combined to allow for more accurate read alignment with strobealign for the

shortest read lengths. While the combined results were obtained as a proof-of-concept by running strobealign twice, more efficient solutions could be implemented, as discussed in future work.

References

[1] Robert Edgar. Syncmers are more sensitive than minimizers for selecting conserved k-mers in biological sequences. *PeerJ*, 9:e10805–e10805, Feb 2021. 33604186[pmid].

[2] Can Firtina, Jisung Park, Mohammed Alser, Jeremie S Kim, Damla Senol Cali, Taha Shahroodi, Nika Mansouri Ghiasi, Gagandeep Singh, Konstantinos Kanellopoulos, Can Alkan, and Onur Mutlu. Blend: a fast, memory-efficient and accurate mechanism to find fuzzy seed matches in genome analysis. *NAR Genom Bioinform*, 5(1):lqad004, Mar 2023.

[3] Heng Li. Aligning sequence reads, clone sequences and assembly contigs with BWA-MEM, 2013.

[4] Benjamin Dominik Maier and Kristoffer Sahlin. Entropy predicts sensitivity of pseudo-random seeds. *Genome Research*, 2023.

[5] Sergey Nurk, Sergey Koren, and Arang Rhie et al. The complete sequence of a human genome. *Science*, 376(6588):44–53, 2022.

[6] Kristoffer Sahlin. Effective sequence similarity detection with strobemers. *Genome research*, 31(11):2080–2094, Nov 2021. 34667119[pmid].

[7] Kristoffer Sahlin. Strobealign: flexible seed size enables ultra-fast and accurate read alignment. *Genome Biology*, 23(1):260, 2022.

4.1 Figures

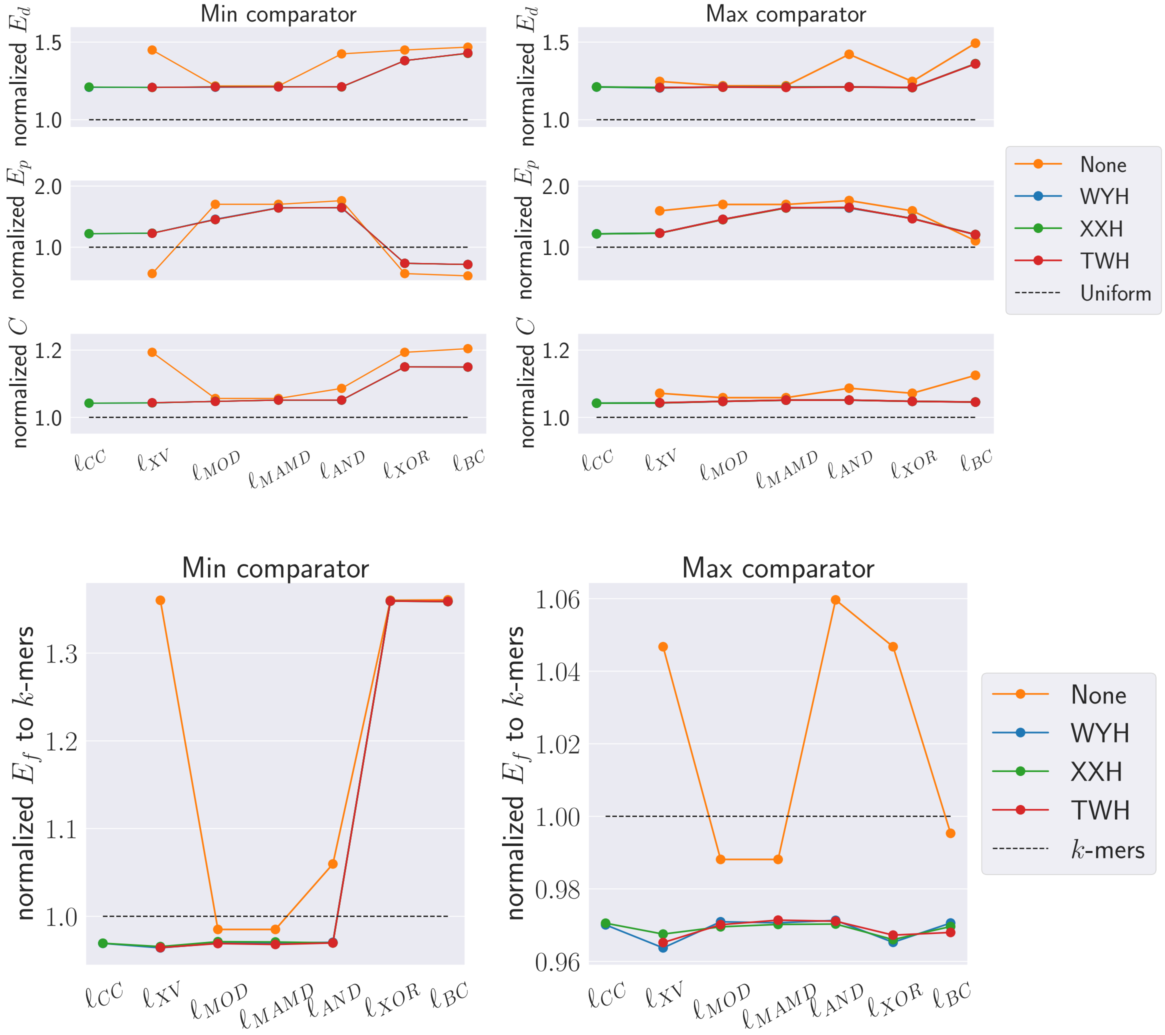

Figure S1: **SIM dataset**. **Upper panel:** Results for metrics E_d (upper panels), E_p (middle panels), and C (lower panels) for randstrobes with parameter settings ($n = 2, l = 20, w_{min} = 21, w_{max} = 100$) for the repetitive sequence dataset. The x-axis shows the different linking methods, and the min and max comparators are shown in left and right panels, respectively. We have normalized the values with a near ideal result produced by simulating strobes uniformly at random in the window with `rand()`. Therefore, a value of 1.0 indicates best possible outcome (indicated by black dashed line). The hash functions WYH, XXH and TWH achieve near identical results, hence only the last plotted line (TWH) is visible. **Lower panel:** E-hits of seed hash values for randstrobes with parameters ($n = 2, l = 20, w_{min} = 21, w_{max} = 100$). The values have been normalized with the E-hits of k-mers of size 40 (dashed black line). Lower value is better.

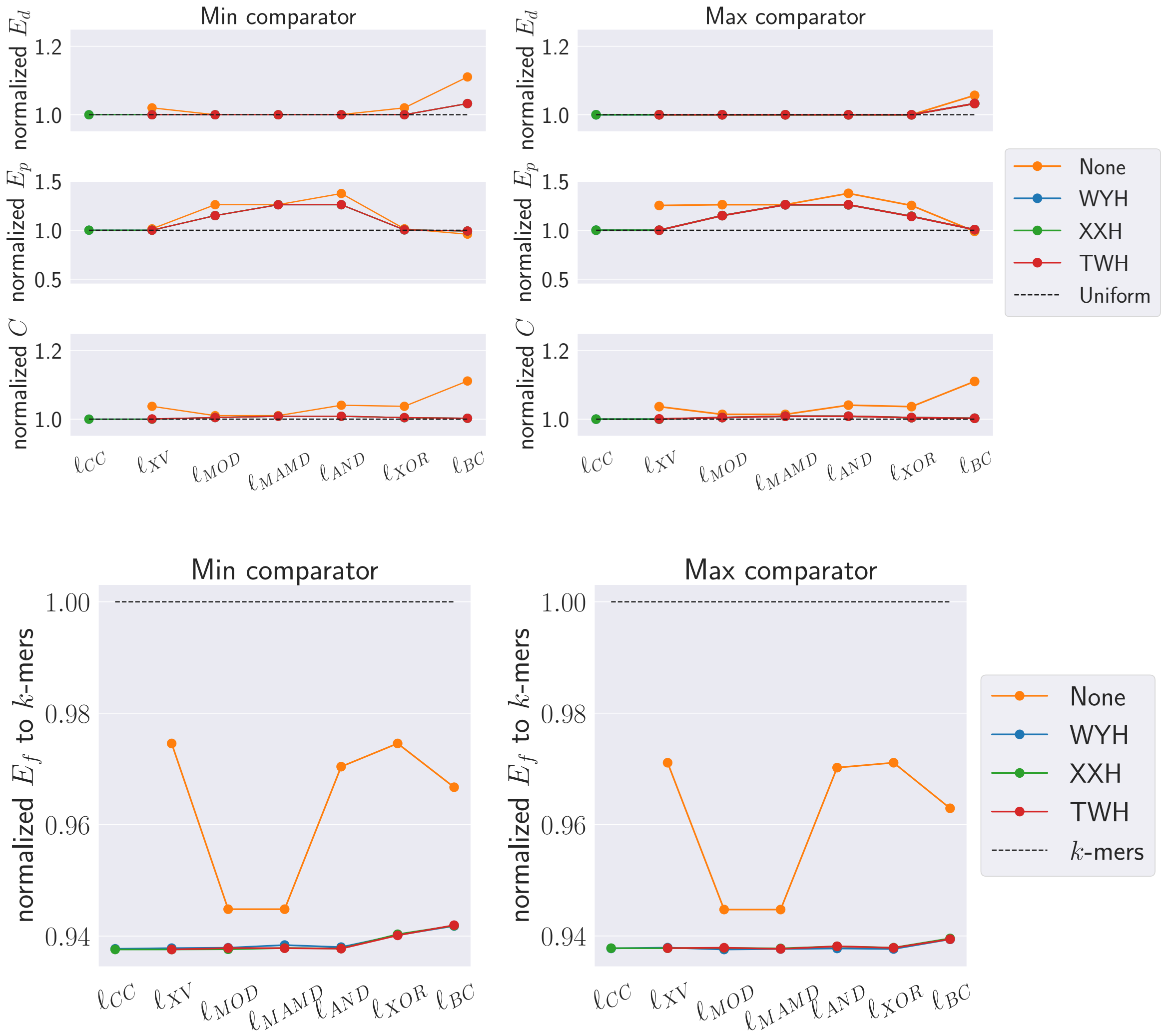

Figure S2: **E20 dataset**. **Upper panel:** Results for metrics E_d (upper panels), E_p (middle panels), and C (lower panels) for randstrobes with parameter settings ($n = 2, l = 20, w_{min} = 21, w_{max} = 100$) for the repetitive sequence dataset. The hash functions WYH, XXH and TWH achieve near identical results, hence only the last plotted line (TWH) is visible. **Lower panel:** E-hits of seed hash values for randstrobes with parameters ($n = 2, l = 20, w_{min} = 21, w_{max} = 100$). The values have been normalized with the E-hits of k-mers of size 40 (dashed black line).

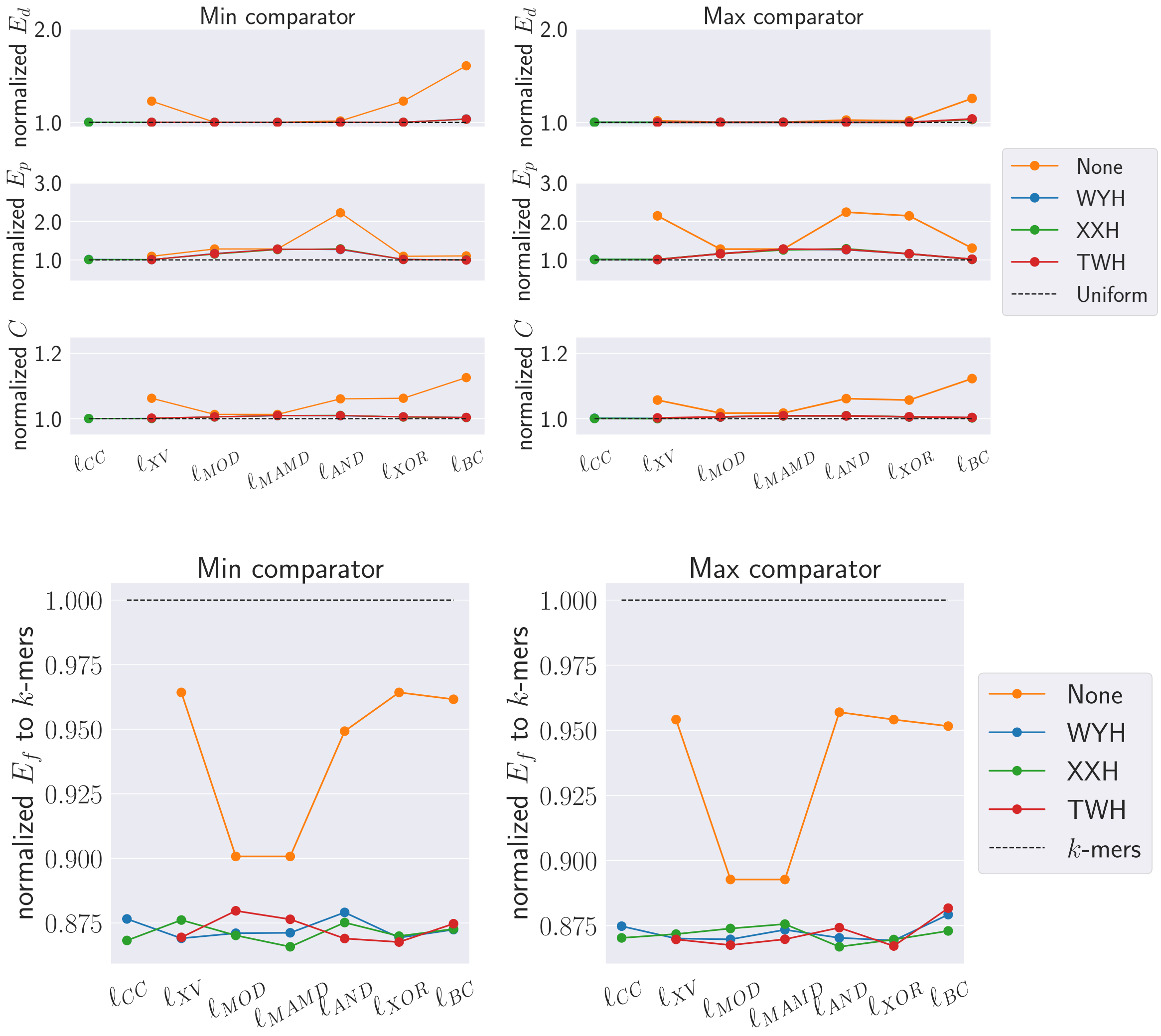

Figure S3: **ChrY dataset**. **Upper panel:** Results for metrics E_d (upper panels), E_p (middle panels), and C (lower panels) for randstrobes with parameter settings ($n = 2, l = 20, w_{min} = 21, w_{max} = 100$) for the repetitive sequence dataset. The hash functions WYH, XXH and TWH achieve near identical results, hence only the last plotted line (TWH) is visible. **Lower panel:** E-hits of seed hash values for randstrobes with parameters ($n = 2, l = 20, w_{min} = 21, w_{max} = 100$). The values have been normalized with the E-hits of k-mers of size 40 (dashed black line).

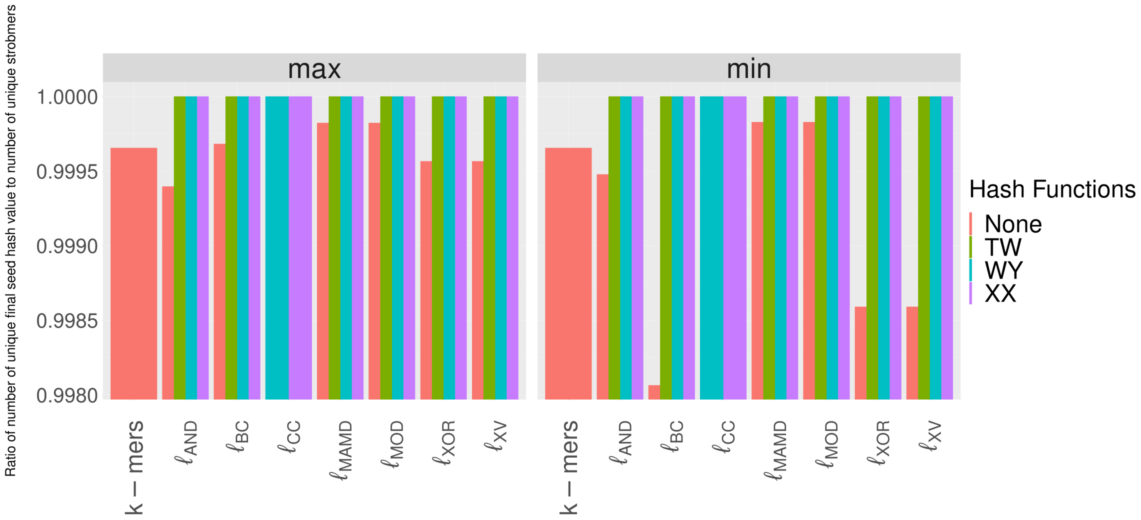

Figure S4: Ratio of number of unique final seed hash value to number of unique strobemers for $(n = 2, l = 20, w_{min} = 21, w_{max} = 100)$.

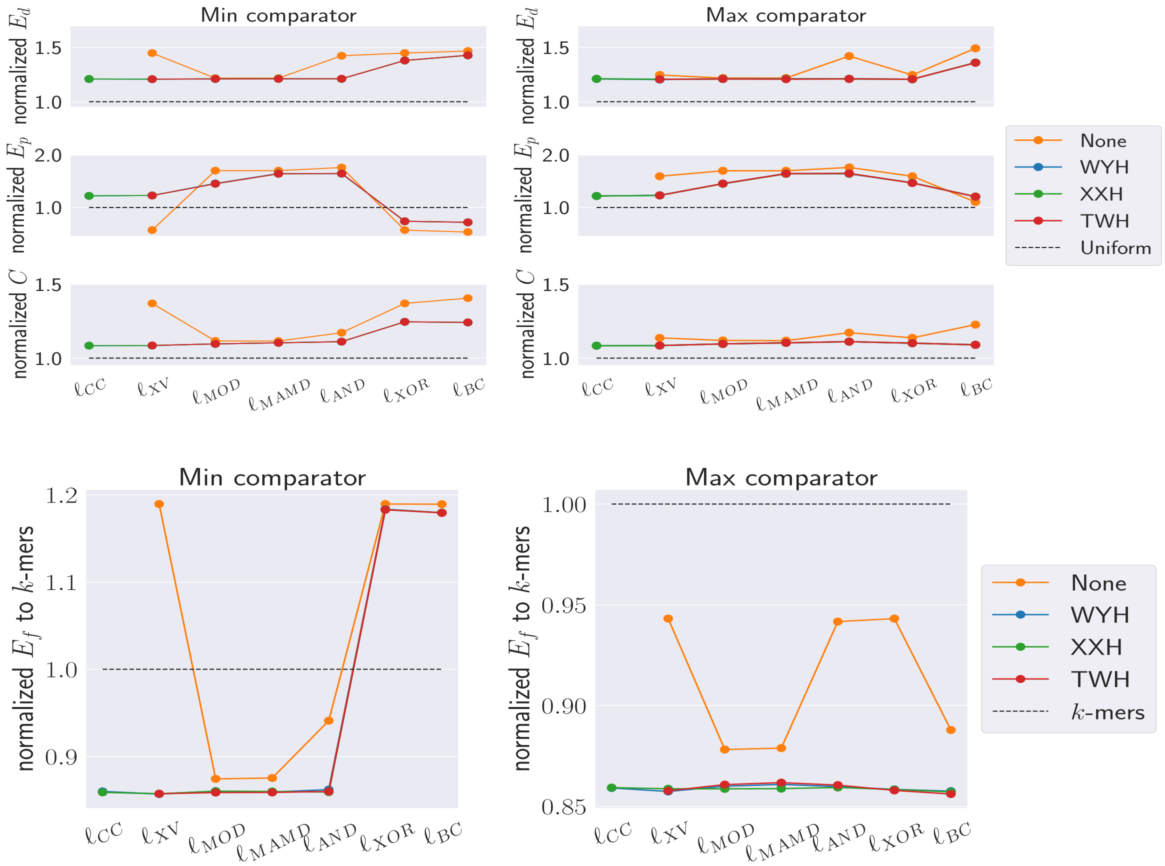

Figure S5: **Benchmark with three strobes on SIM**. **Upper panels:** Results for metrics E_d, E_{p3}, and C for randstrobes with parameter settings $(n = 3, l = 20, w_{min} = 21, w_{max} = 100)$ for the repetitive sequence dataset. The hash functions WYH, XXH and TWH achieve near identical results, hence only the last plotted line (TWH) is visible. **Lower panels:** E-hits of final seed hash values for randstrobes with three strobes with parameter settings $(n = 3, l = 20, w_{min} = 21, w_{max} = 100)$ compared to k-mers with $k = 60$

4.2 Tables

Table S1: Strobealign accuracy results (% of total read pairs) when mapping paired-end reads using the min comparator, max comparator, and when selecting the best read alignment between the two versions (combined). The diff column shows the percent point difference in accuracy between combined and min-comparator only, and the rightmost column indicate the difference of the combined results to that of BWA, the overall most accurate aligner in the benchmark in [7]. Negative values indicate that strobealign has higher accuracy.

dataset (genome-read length)	min comparator	max comparator	combined	diff	combined diff to BWA
drosophila-50	90.1890	90.1542	90.4216	+0.2326	+0.0901
drosophila-75	91.6441	91.6422	91.6818	+0.0377	-0.0233
drosophila-100	92.3879	92.3948	92.4168	+0.0289	-0.0207
drosophila-150	93.2112	93.2220	93.2302	+0.0190	-0.0350
drosophila-200	93.5209	93.5309	93.5347	+0.0138	-0.0617
drosophila-300	95.3608	95.3681	95.3734	+0.0126	-0.0425
drosophila-500	95.6936	95.7124	95.7132	+0.0196	-0.0774
CHM13-50	90.6350	90.5854	91.0971	+0.4621	+0.5227
CHM13-75	92.5158	92.5197	92.6462	+0.1304	+0.2038
CHM13-100	93.2198	93.2153	93.3182	+0.0983	+0.1367
CHM13-150	94.1404	94.1486	94.2101	+0.0698	+0.0543
CHM13-200	94.4340	94.4397	94.4870	+0.0530	+0.0241
CHM13-300	95.6266	95.6271	95.6779	+0.0512	+0.0796
CHM13-500	95.9505	95.9555	96.0153	+0.0648	+0.0523
rye-50	69.1402	68.9105	71.1016	+1.9613	+2.3892
rye-75	80.5345	80.4464	81.5855	+1.0511	+1.4724
rye-100	85.6483	85.6312	86.4098	+0.7615	+0.9966
rye-150	90.2038	90.2065	90.6332	+0.4295	+0.4415
rye-200	91.4773	91.4661	91.7506	+0.2733	+0.1812
rye-300	94.5574	94.5816	94.6644	+0.1070	+0.1012
rye-500	95.1326	95.1618	95.2114	+0.0787	+0.0374
maize-50	71.4703	71.3223	73.0630	+1.5927	+1.6149
maize-75	82.1255	82.0405	82.9049	+0.7794	+0.7763
maize-100	87.1317	87.1404	87.7111	+0.5793	+0.5152
maize-150	91.6731	91.6841	91.9923	+0.3191	+0.1784
maize-200	92.9204	92.9328	93.1210	+0.2005	+0.0883
maize-300	96.7084	96.7183	96.8246	+0.1163	+0.0332
maize-500	97.2899	97.2962	97.4021	+0.1122	-0.0025

Table S2: Strobealign accuracy results (% of total reads) when mapping single-end reads using the min comparator, max comparator, and when selecting the best read alignment between the two versions (combined). The diff column shows the difference between combined and min comparator only, and the rightmost column indicate the difference of the combined results to that of BWA, the overall most accurate aligner in the benchmark in [7].

dataset (genome-read length)	min comparator	max comparator	combined	diff	combined diff to BWA
drosophila-50	82.5118	82.3825	84.2688	+1.7570	+2.5606
drosophila-75	87.9944	87.9556	88.2902	+0.2958	+0.2191
drosophila-100	89.2585	89.2853	89.4715	+0.2130	+0.1209
drosophila-150	90.9438	90.9455	91.0235	+0.0797	+0.0184
drosophila-200	91.9501	91.9644	91.9927	+0.0426	+0.0286
drosophila-300	93.2169	93.2451	93.2396	+0.0227	+0.0067
drosophila-500	94.5768	94.5895	94.6109	+0.0341	+0.0162
CHM13-50	81.6910	81.5544	83.6503	+1.9593	+2.9080
CHM13-75	88.9220	88.9050	89.4662	+0.5442	+0.6695
CHM13-100	90.6392	90.6112	91.0038	+0.3646	+0.3714
CHM13-150	92.3990	92.3885	92.5668	+0.1678	+0.1467
CHM13-200	93.2258	93.2383	93.3355	+0.1097	+0.0965
CHM13-300	94.1503	94.1331	94.2176	+0.0673	+0.1184
CHM13-500	95.0598	95.0737	95.1460	+0.0862	+0.1218
rye-50	44.6869	44.4947	46.1874	+1.5005	+2.1252
rye-75	60.2210	60.0863	61.3118	+1.0908	+1.5198
rye-100	69.3358	69.3736	70.4965	+1.1607	+1.5197
rye-150	80.4388	80.3968	81.3871	+0.9483	+0.8698
rye-200	85.9181	85.8857	86.6284	+0.7103	+0.6255
rye-300	90.5230	90.5150	90.9841	+0.4611	+0.5704
rye-500	93.5070	93.5344	93.7914	+0.2844	+0.3686
maize-50	47.4174	47.2713	48.8452	+1.4278	+2.0588
maize-75	61.9111	61.8251	62.8084	+0.8973	+1.1361
maize-100	70.5000	70.4634	71.4263	+0.9263	+1.0928
maize-150	81.1708	81.1807	81.9200	+0.7492	+0.5582
maize-200	86.7010	86.7061	87.2567	+0.5557	+0.3617
maize-300	91.8729	91.8704	92.2597	+0.3868	+0.3108
maize-500	95.4025	95.4229	95.7271	+0.3246	+0.2333

Table S3: Strobealign accuracy results (% of total reads) when mapping single-end reads using the min comparator, max comparator, and when adding the reads that were mapped only with the max comparator to the mapping results with the min comparator (combined). The diff column shows the difference between combined and min comparator only.

dataset (genome-read length)	min comparator	max comparator	combined	diff
drosophila-50	82.5118	82.3825	84.1942	+1.6824
drosophila-75	87.9944	87.9556	88.2319	+0.2375
drosophila-100	89.2585	89.2853	89.4176	+0.1591
drosophila-150	90.9438	90.9455	90.9823	+0.0385
drosophila-200	91.9501	91.9644	91.9590	+0.0089
drosophila-300	93.2169	93.2451	93.2170	+0.0001
drosophila-500	94.5768	94.5895	94.5769	+0.0001
CHM13-50	81.6910	81.5544	83.1962	+1.5052
CHM13-75	88.9220	88.9050	89.1354	+0.2134
CHM13-100	90.6392	90.6112	90.7793	+0.1401
CHM13-150	92.3990	92.3885	92.4377	+0.0387
CHM13-200	93.2258	93.2383	93.2328	+0.0070
CHM13-300	94.1503	94.1331	94.1504	+0.0001
CHM13-500	95.0598	95.0737	95.0598	+0.0000
rye-50	44.6869	44.4947	45.2202	+0.5333
rye-75	60.2210	60.0863	60.2857	+0.0647
rye-100	69.3358	69.3736	69.3846	+0.0488
rye-150	80.4388	80.3968	80.4509	+0.0121
rye-200	85.9181	85.8857	85.9201	+0.0020
rye-300	90.5230	90.5150	90.5230	+0.0000
rye-500	93.5070	93.5344	93.5070	+0.0000
maize-50	47.4174	47.2713	48.0640	+0.6466
maize-75	61.9111	61.8251	61.9915	+0.0804
maize-100	70.5000	70.4634	70.5609	+0.0609
maize-150	81.1708	81.1807	81.1898	+0.0190
maize-200	86.7010	86.7061	86.7042	+0.0032
maize-300	91.8729	91.8704	91.8729	+0.0000
maize-500	95.4025	95.4229	95.4025	+0.0000
